# Clinical characterization of Lamb-Shaffer syndrome: a case report and literature review

**DOI:** 10.1186/s12920-023-01448-4

**Published:** 2023-02-09

**Authors:** Guo-qing Zhu, Ping Dong, Dong-yun Li, Chun-chun Hu, Hui-ping Li, Ping Lu, Xue-xia Pan, Lin-lin He, Xiu Xu, Qiong Xu

**Affiliations:** 1 Child Health Care Department, Children’s Hospital of Fudan University, Shanghai, China; 2 Pediatric Department, Binzhou People’s Hospital, Binzhou, Shandong China; 3 Pediatric Department, Suining Central Hospital, Suining, Sichuan China

**Keywords:** Clinical characterization, Lamb-Shaffer syndrome, *SOX5*

## Abstract

**Background:**

Lamb-Shaffer syndrome (LAMSHF, MIM 616,803) is a rare neurodevelopmental disorder due to haploinsufficiency of *SOX5*. Furthermore, studies about the clinical features of LAMSHF patients with same allele of c.1477C > T (p. R493*) are very limited.

**Case presentation:**

We analyzed the phenotypes of one of our cases and two previously reported cases with c.1477C > T (p. R493*), and reviewed the correlating literature. A de novo heterozygous variation c.1477C > T (p. R493*) in *SOX5* was identified in a 4 years and 2 months old boy with global development delay by trio-based whole exome sequencing. We compared our case and previously 2 cases reported with recurrent variation, the overlapping clinical features are global developmental delay or intellectual disability, language delay and scoliosis, but their other clinical characteristics are different.

**Conclusions:**

This study suggests that the clinical features of LAMSHF patients with recurrent variations in the *SOX5* gene are different. It is suggested that the LAMSHF-related *SOX5* gene should be screened and included as one of the candidate genes for neurodevelopmental disorders of unknown etiology.

## Background

Lamb-Shaffer syndrome (LAMSHF, MIM 616,803) is a neurodevelopmental disorder caused by genetic alterations due to haploinsufficiency of the *SOX5* gene (SRY-related high-mobility-group box5, MIM 604,975) [1, 3]. LAMSHF is clinically characterized by global developmental delay (GDD), intellectual disability (ID), language delay, behavioral disturbances including autistic features, and dysmorphic facial features [1–3]. The *SOX5* gene is located on chromosome 12p12.1, and is the key gene encoding a transcription factor regulating cell fate and differentiation during neural development [1, 4]. Haploinsufficiency of *SOX5* is mostly caused by deletions and point variation [1, 5, 6]. To date, only 74 LAMSHF patients have been reported worldwide [1–3, 6–13]. There is no specific treatment for LAMSHF. Life expectancy of individuals with LAMSHF does not to be affected. The genotype–phenotype correlations are still not clear [6]. Here, we report the genetic and clinical manifestations of a patient with LAMSHF and compare them with 2 previously reported cases with recurrent variation. To extend the clinical characteristics and genotypes of Lamb-Shaffer syndrome, related literature was also reviewed and summarized.

## Case presentation

The clinical data of the patient with LAMSHF were analyzed retrospectively. The developmental level was assessed using the Griffiths Development Scales-Chinese (GDS-C), including subscales of locomotor (A), personal-social (B), language (C), eye-hand coordination (D) and performance (E). We used trio-based whole exome sequencing (WES) to analyze the genes of the case. Sanger sequencing was performed to validate the variations and determine their parental origin. The variants were classified as “pathogenic” or “likely pathogenic” or “benign” or “likely pathogenic” according to the variant interpretation guidelines of the American College of Medical Genetics and Genomics [5]. Previous cases were compared, and related literature was reviewed.

**Fig. 1:**
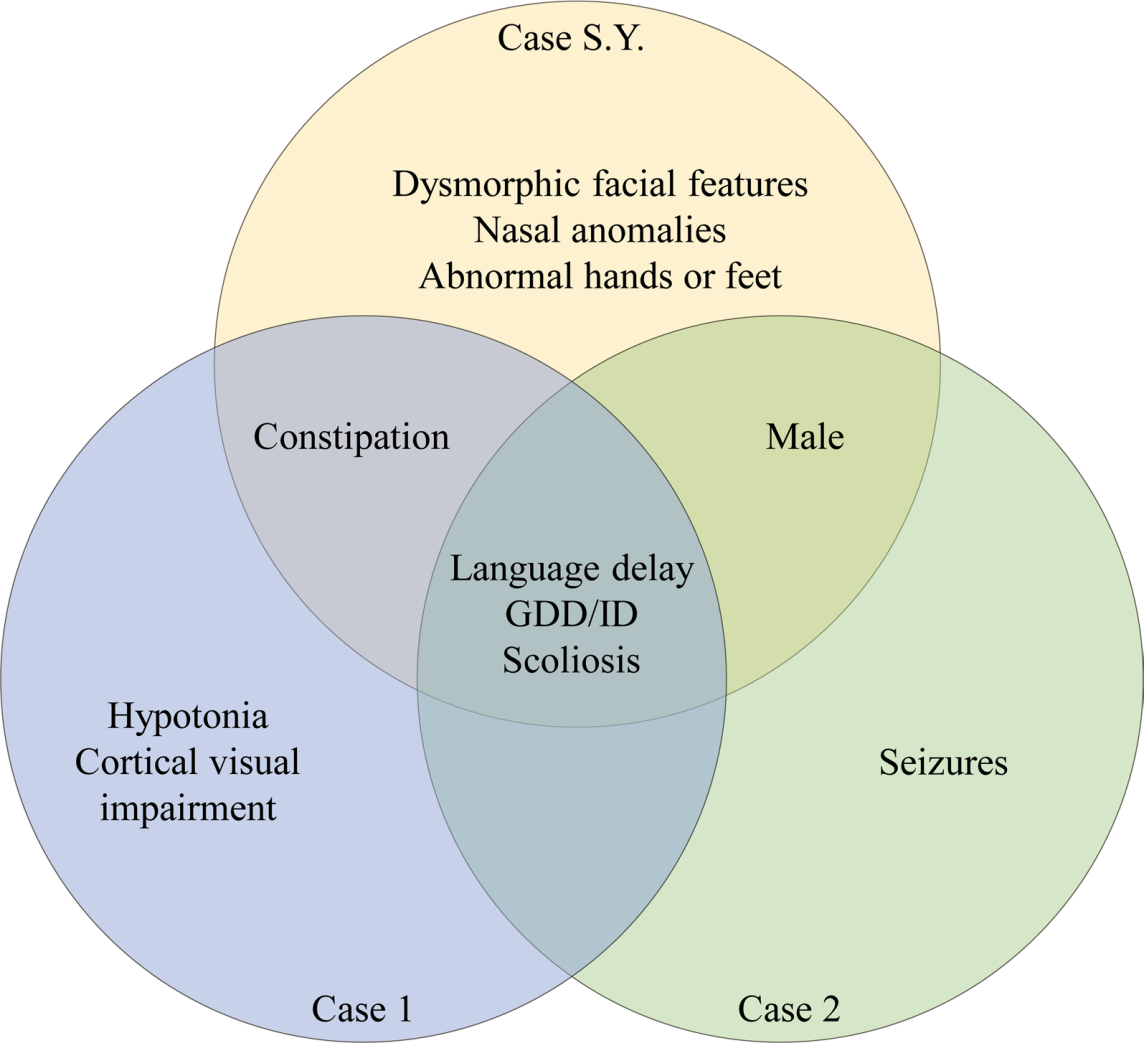
Description of the clinical features of our case S.Y. and previously reported two cases with the same allele of *SOX5* c.1477C > T (p. R493 *)

The patient was male, the first child, born full-termly, and his parents were healthy and nonconsanguineous. The birth weight was 3750 g, and the length was 52.0 cm. He was able to stand alone at 12 months old and walk without support at 16 months old. The patient visited our clinic at the age of 50 months old, and the weight was 16.0 kg ( − 0.35 SD), the length was 105.5 cm (+ 0.26 SD) and the head circumference was 50.0 cm ( − 0.20 SD). He started to vocalize for approximately 18 months, and he could only speak 2-word phrases instead of sentences at admission. He had scoliosis and constipation. His facial features included a flat nasal bridge and overlapping toes and toe contractures of the feet. Family history was negative for any neurodevelopmental disorder or genetic disorders. A detailed description of the clinical features of our case S.Y. and previously reported cases with *SOX5* variants, and comparisons with other patients are shown in Table 1 and Fig. 1.

**Table 1:** Comparison of clinical features of the case S.Y. and previously cases with the *SOX5* variants

	Case S.Y. with *SOX5* c.1477C > T (p.R493*)	Case 1 with *SOX5* c.1477C > T (p.R493*)	Case 2 with *SOX5* c.1477C > T (p.R493*)	Previous cases with other *SOX5* nonsense variants (n = 13)	Previous cases with other *SOX5* variants (n = 72)
Age of diagnosis (years)	4.2	3	20	13.7 ± 11.3	12.2 ± 11.9
Sex	Male	Female	Male	Male (5/12) Female (7/12)	Male (38/71) Female (33/71)
Language delay	+	+	+	13/13	64/66
GDD/ID	+	+	+	11/12	62/66
Dysmorphic facial features	+	NA	NA	2/2	54–56/71
Nasal anomalies	+	NA	NA	0/1	20–22/71
Teeth anomalies	−	NA	NA	1/1	7–9/71
Small jaw	−	NA	NA	1/1	6–8/71
Blue sclerae	−	NA	NA	0/1	0–2/71
Behavioral disturbances	−	−	−	8/10	37/49
ASD	−	−	−	2/13	10/69
Scoliosis	+	+	+	3/13	10/71
Abnormal hands or feet	+	−	−	2/13	17/71
Joint hyperlaxity	−	−	−	0/13	4/71
Fused vertebrae	−	−	−	0/13	3/71
Constipation	+	+	−	0/2	8/71
Hypotonia	−	+	−	8/13	36/70
Seizures	−	−	+	2/12	17/70
Microcephaly	−	−	−	1/13	11/70
Gait ataxia	−	−	−	1/13	7/71
Tetrapyramidal syndrome	−	−	−	2/13	2/71
Strabismus	−	−	−	7/13	29/71
Optic atrophy	−	−	−	5/13	8/71
Myopia	−	−	−	2/13	6/71
Amblyopia	−	−	−	0/13	2/71
Cortical visual impairment	−	+	−	0/13	0/71

*NA* Not available, *GDD* Global developmental delay, *ID* Intellectual disability, *ASD* Autism spectrum disorder

The results of Locomotor (A), Personal-social (B), Language (C), Eye-hand co-ordination (D) and Performance (E) of GDS-C were 5th pencentile, <1st pencentile, < 1th pencentile, < 1th pencentile and 2.5th pencentile, respectively. X-ray examination of the spine showed mildly scoliosis. Brain magnetic resonance imaging (MRI) showed bilateral ventricular dilatation. His thyroid function was normal. Hearing test was passed. Eye exams were normal. Electroencephalogram (EEG), cardiac sonography and abdominal sonography examinations were also normal.

**Fig. 2:**
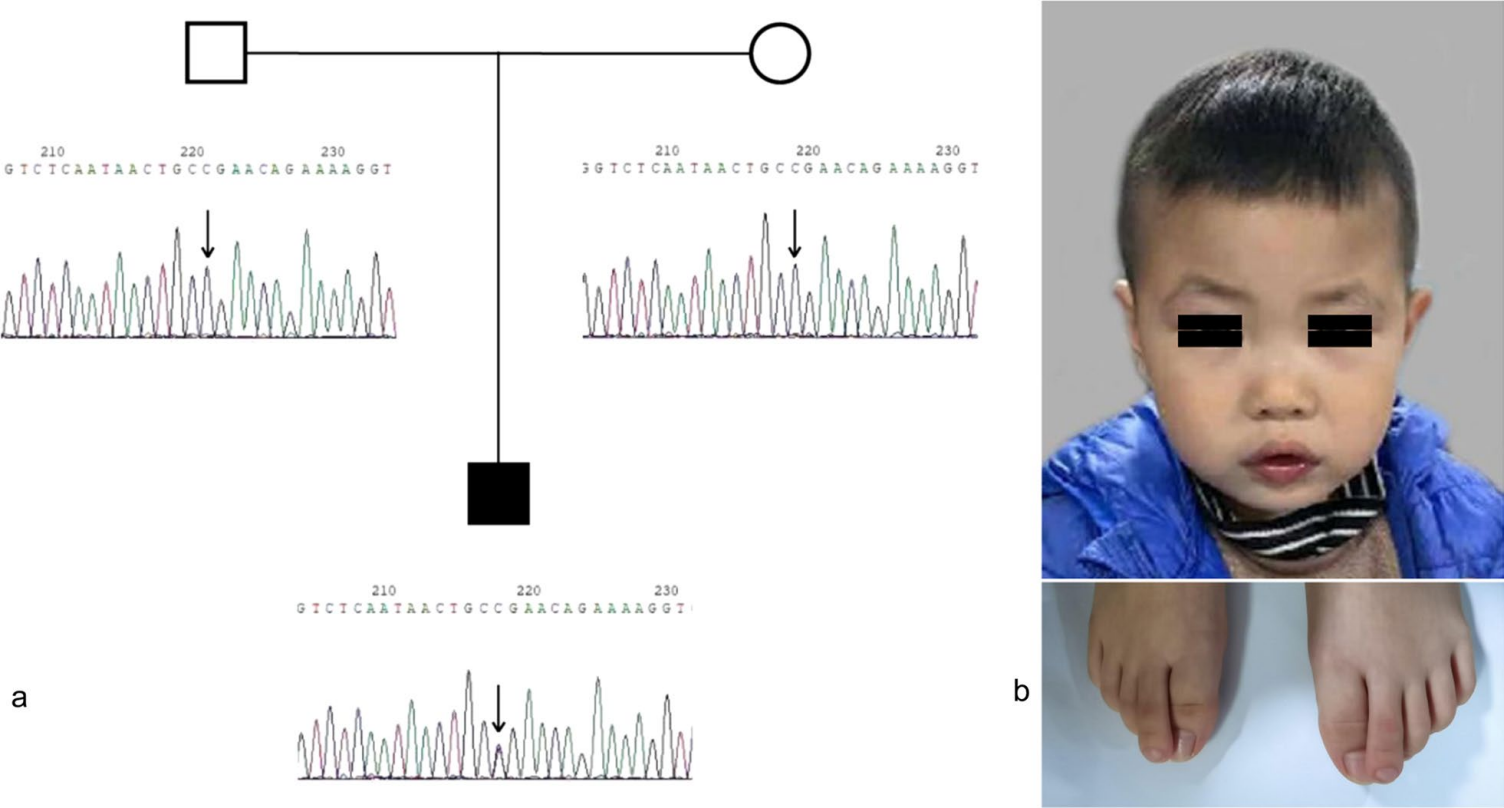
S.Y.with a de novo heterozygous *SOX5* variant. **a** Sanger sequencing confirmation for c. 1477C > T *SOX5* variant in the proband but absent in both parents. **b** The figure above shows flat nasal bridge. The figure below shows overlapping toes and toe contractures

**Fig. 3:**
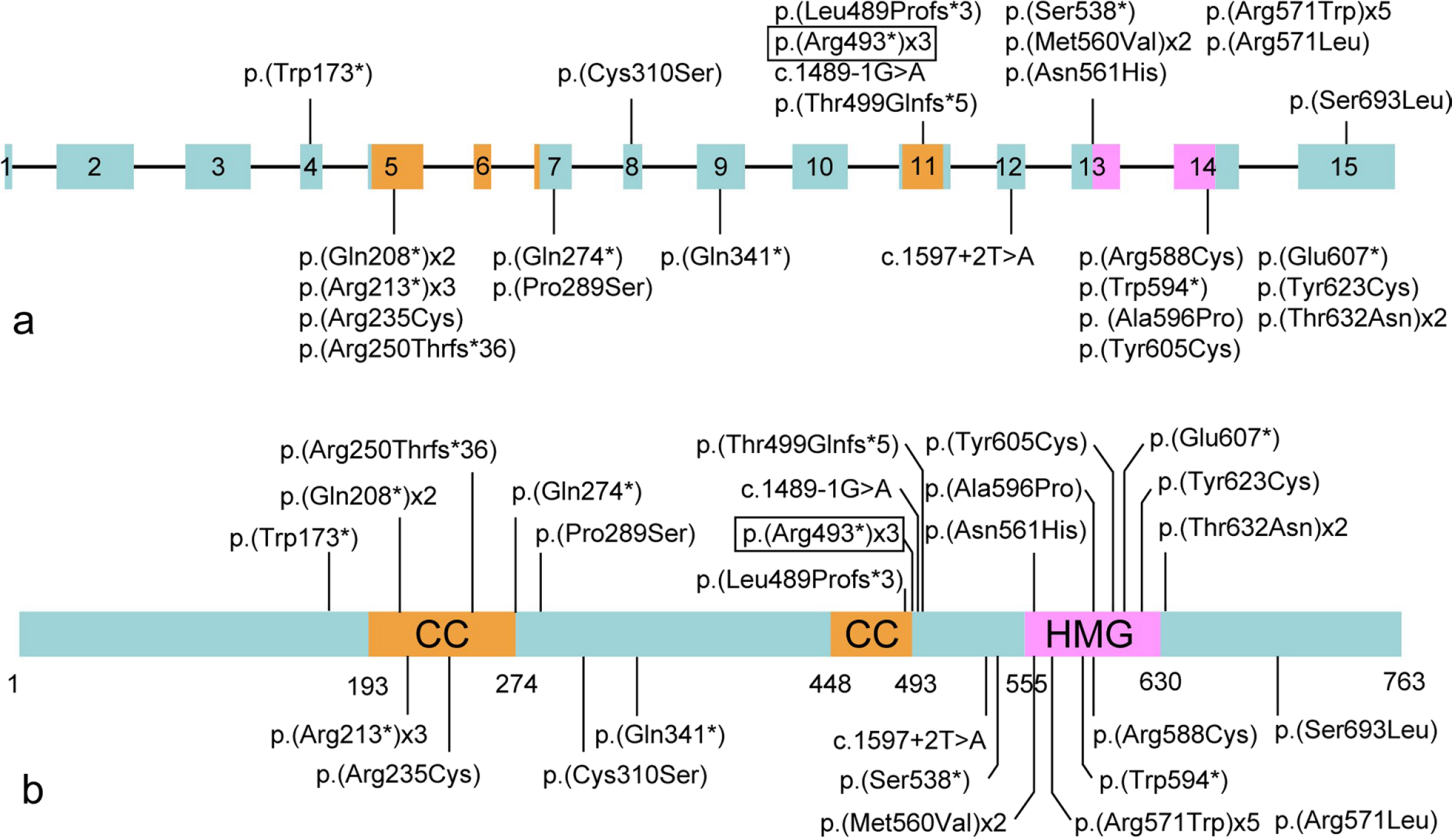
Genetic location of the *SOX5* point variants associated with LAMSHF identified to date. **a** Boxes 1 to 15, coding exons of isoform NM_006940. The variant reported in this case showed a square frame. We used bars to report the variants, and " × " represents the number of cases. The size of exon and intron is not proportional. **b** Distribution of amino acid changes related to the protein domains CC (coiled-coil) and HMG (high-mobility-group). Protein and domain residue boundaries are indicated underneath the schematic

WES identified a de novo heterozygous variant c.1477C > T of *SOX5* and confirmed it by Sanger sequencing (Fig. 2). *SOX5* c.1477C > T (p. R493*) was a nonsense variant and was classified as pathogenic. Other pathogenicity point variants previously reported are also shown as comparisons (Fig. 3).

## Discussion and conclusions

Case S.Y. in our study was the third case with the same allele of *SOX5* c.1477C > T (p. R493 *). Previously reported case 1 was a 3-year-old female with global developmental delay, hypotonia, cortical visual impairment, scoliosis, ear tubes, constipation and supernumerary nipple. The other previously reported case 2 was a 20-year-old male with intellectual disability, epilepsy and scoliosis and inability to speak [6]. The 3 cases with recurrent *SOX5* variant shared similar clinical manifestations, such as global developmental delay or intellectual disability, language delay and scoliosis. Particularly, our case S.Y. exhibited flat nasal bridges, overlapping toes, toe contractures and constipation.

The common and different manifestations may suggest heterogeneous clinical phenotypes underlying the same genotype. Previous cases with other recurrent point variants of the *SOX5* gene, such as p. Gln208* 2 cases, p. Arg213* 3 cases, p. Met560Val 3 cases, p. Arg571Trp 5 cases, p. Thr632Asn 2 cases also demonstrated different clinical manifestations [6, 8]. For example, 5 patients have been reported with the variant p.Arg571Trp. A total of 3/5 showed hypotonia, 1/5 showed microcephaly, and the most severe patient showed seizures, strabismus and joint hyperlaxity. The differences in the phenotypes may be explained by the additional variations associated with the same gene, however in those cases other implicated genes are not available for us. Other than point variants, patients with the same deletion alteration of *SOX5* also exhibited different clinical manifestations. For instance, 2 patients with the same exon 8–10 deletion both had seizures, but only one of them showed fused vertebrae, subaortic ventricular septal defects and pulmonary stenosis [6]. Regarding the remaining 72 cases with LAMSHF, language development delay was present in almost all of the cases [1–3, 6–13]. Hypotonia, ophthalmic features, and scoliosis are also commonly reported [1, 6, 9]. Approximately 1/4 of reported cases showed seizures with a positive response to medication, followed by a benign course [7, 12].

The incomplete penetrance suggests that *SOX5* haploinsufficiency may manifest differently in similar genetic backgrounds. One possible reason could be that some variants of *SOX5* retain partial activity [14]. We hypothesize that there could be environmental influences, epigenetics, life style and genetic background of other genetic variation contributing to incomplete penetrance and variable expressivity. Longitudinal studies with larger sample sizes as well as molecular and histological studies on animal models are highly recommended for the elucidation of the pathological mechanisms of *SOX5* partial activity.

Notably, all of the LAMSHF cases were caused by heterozygous variations*.* The majority of the cases were reported with *SOX5* deletion and point variation, including 18 cases with missense variation (12 types), 13 cases with nonsense variation (9 types), and 3 cases with shift variation (3 types) [6, 7, 11, 13]. Our case was tested with the nonsense point variant c.1477C > T (p. R493 *), which will lead to premature termination and absence of the HMG domain in the translated protein.

The HMG domain is the core functional domain of the *SOX* protein family, which mediates DNA binding and bending and regulates the expression of downstream developmental genes [15]. For humans, half of the pathogenic variants of the *SOX* genes result in developmental disorders [14]. The *SOX5* gene plays an important role in the development of the nervous system, chondrogenesis and other developmental pathways [4]. According to the amino acid sequences of the HMG domain, the *SOX* protein is divided into groups A-H [16]. *SOX5* belongs to the *SOXD* group, which consists of a unique long N-terminal sequence containing a population-specific coiled-coil (CC) domain [17]. The CC domain mediates *SOXD* protein dimerization, thereby preferentially binding to pairs of DNA recognition sites [17].

Moreover, *SOX5* can produce at least 5 transcript isoforms through promoter expression, start site alternative and precursor messenger RNA (premRNA) splicing [18]. The variant of our case was on exon 11, which leads to haploinsufficiency of 3 transcript isoforms *L1-SOX5* (NM_006940), *L2-SOX5* (NM_152989) and *S-SOX5* (NM_178010). The isoform *L1-SOX5* is composed of exons 1–15 encoding the protein. The isoform *L2-SOX5* consists of a 5’ noncoding protein sequence, and exons 2–15 encode the protein. Both *L1-SOX5* and *L2-SOX5* are the predominant brain isoforms and play an important role in the formation and functional differentiation of the fetal brain [11, 18]. The shortest isoform *S-SOX5* is composed mainly of exon 10–15 encoding protein and is testes-specific [19]. It may play an important role in the formation and function of motor cilia in the brain, lungs, testes and spermatozoa [19]. In our case, we observed developmental delay and language delay, while the effects on the lung and testis were further followed up.

In conclusion, the current study reported one LAMSHF case with the *SOX5* gene variant. The case shared not only similar but also specific clinical features compared with previous cases, which helped to extend the phenotypic spectrum.

The differences in the phenotypes may be explained by the additional variations or incomplete penetrance and variable expressivity. LAMSHF may affect multiple systems, especially language and intellectual development. However, the diagnosis of LAMSHF requires genetic testing to confirm. Thus, it is suggested that the LAMSHF-related *SOX5* gene should be screened and included as one of the candidate genes for neurodevelopmental disorders of unknown etiology. Systematic developmental studies with follow-up and larger sample sizes should be performed to investigate the function of *SOX5* in humans and provide more evidence for clinical decisions. Future studies on LAMSHF patients with same variants iPSC derived neurons could be conducted as different phenotypic variations were observed. This could lead to better understand the disorder's molecular pathogenesis, as well as looking into the potential causes of the variation seen, including genetic modifiers and polygenic factors.

## Data Availability

The datasets used and/or analysed during the current study are available from the corresponding author on reasonable request. And the genetic testing data has been uploaded to ClinVar [VCV000695081.6].

## Abbreviations

LAMSHF: Lamb-Shaffer syndrome
*SOX5*: SRY-related high-mobility-group box5
GDD: Global developmental delay
ID: Intellectual disability
GDS-C: Griffiths development scales-Chinese
WES: Whole exome sequencing
NA: Not available
ASD: Autism spectrum disorder
MRI: Magnetic resonance imaging
EEG: Electroencephalogram
CC: Coiled-coil
HMG: High-mobility-group
